# The Changing Landscape of Peritoneal Dialysis in America: A Facility-Level Analysis of Growth

**DOI:** 10.1016/j.xkme.2026.101391

**Published:** 2026-05-08

**Authors:** Ankur D. Shah, Sandipan Shringi, Afzal Ariff, Cara J. Sammartino, Christina A. Raker, Susie L. Hu

**Affiliations:** 1 Warren Alpert Medical School of Brown University, Providence, Rhode Island; 2 Division of Kidney Disease and Hypertension, Department of Medicine, Rhode Island Hospital, Providence, Rhode Island; 3 Brown University, Providence, Rhode Island; 4 Johnson and Wales University, Providence, Rhode Island; 5 Lifespan Biostatistics, Epidemiology, Research Design, Informatics Core, Rhode Island Hospital, Providence, Rhode Island

**Keywords:** Peritoneal dialysis, home dialysis, dialysis facility, end-stage kidney disease, health care delivery

## Abstract

**Rationale & Objective:**

Peritoneal dialysis (PD) use has expanded nationwide, yet it is unclear whether this growth reflects more facilities initiating PD programs, expansion of existing programs, or both. We sought to describe national trends in PD program adoption and program size and to assess whether these patterns differed across facility and geographic characteristics.

**Study Design:**

Retrospective serial cross-sectional study.

**Setting & Participants:**

All Medicare-certified dialysis facilities in the United States from 2010-2021.

**Exposure:**

Calendar year.

**Outcomes:**

(1) Whether a facility treated ≥1 PD patient; (2) PD census among facilities with active PD programs.

**Analytical Approach:**

We applied mixed-effects logistic and negative binomial models with random facility effects to estimate changes in PD program presence and the mean census over time. Models were adjusted for region, organizational size, profit status, and urbanicity, and included interaction terms to test for heterogeneity in temporal trends.

**Results:**

The number of US dialysis facilities with active PD programs increased from 38.4%-44.6% between 2010 and 2021, and among those programs, the mean PD census rose from 14.7-18.2. Relative to 2010, facilities in 2021 had higher odds of providing PD (OR, 1.61; 95% CI, 1.35-1.92), and active programs treated substantially more patients (incidence rate ratio, 1.50; 95% CI, 1.45-1.56). Nonprofit and small dialysis organizations showed the greatest likelihood of initiating PD programs, whereas for-profit and large organizations demonstrated stronger census growth. Regional differences were observed, with the West showing the largest increases in program size.

**Limitations:**

Facility-level data prevent examination of patient-level determinants; annual snapshots may miss within-year variation.

**Conclusions:**

From 2010-2021, PD growth reflected both expansion of existing programs and increased program adoption, with substantial differences based on organizational structure. Tailored policy strategies may be needed to support sustained, equitable growth in PD capacity across facility types.

## Plain-Language Summary

Peritoneal dialysis (PD) allows patients with kidney failure to receive treatment at home rather than traveling to a dialysis unit. There has been significant growth in PD in the last decade in the United States, and we wanted to understand how this growth happened. Specifically, were more facilities starting new programs, or were existing programs simply getting bigger and were the types of units that were growing different? We analyzed data from all US dialysis facilities over a decade and found that both more facilities began offering PD and existing programs grew. Growth occurred differently, with smaller and nonprofit organizations more likely to start new programs and larger for-profit organizations more likely to expand existing ones.

Peritoneal dialysis (PD) offers several potential advantages over in-center hemodialysis, including greater flexibility, preserved residual kidney function, and reduced health care costs.^1^^,^^2^ Despite these benefits and policy initiatives aimed at increasing home dialysis use, PD remains underused in the United States compared with many other developed nations.^3^, ^4^, ^5^ Although recent years have seen renewed interest in expanding PD programs, detailed facility-level analyses of PD growth patterns and their determinants remain limited.

PD use has experienced substantial growth over the past decade, with both incident and prevalent PD populations showing marked increases.^2^^,^^6^^,^^7^ The percentage of patients initiating PD has nearly doubled since 2008, with recent data indicating that PD now accounts for ∼15% of the total dialysis population.^2^ Previous studies have largely focused on patient-level factors influencing PD use or aggregate national trends.^2^^,^^6^, ^7^, ^8^, ^9^, ^10^, ^11^ These works have demonstrated regional and rural variations in uptake.^12^, ^13^, ^14^ Understanding how PD programs have evolved at the facility level can provide crucial insights into the transformation of PD care delivery in the United States. It is not known whether growth is occurring through expansion of existing programs, proliferation of new, smaller programs, or both. Organizational characteristics such as profit status, facility size, and geographic location may significantly influence a center’s capacity and willingness to develop robust PD programs, yet the impact of these factors on PD growth trajectories remains poorly understood. Growth patterns across these different facility types and regions can reveal potential disparities in access and identify areas for targeted intervention.

Our study aimed to characterize how the national expansion of PD has manifested at the facility level by examining trends in mean PD census and growth rates from 2010-2021. We analyzed these patterns across different facility characteristics including profit status, organization size, and geographic region to provide a comprehensive picture of how PD programs have evolved over this pivotal decade. Understanding these trends is essential for policymakers, health care systems, and clinicians as they work to further expand access to home dialysis options while maintaining high-quality care.

## Methods

### Study Design and Data Sources

We conducted a retrospective serial cross-sectional study analyzing nationwide trends in PD use from January 1, 2010, through December 31, 2021. This study followed the Strengthening the Reporting of Observational Studies in Epidemiology guidelines for reporting observational studies.^15^ We used data from the Centers for Medicare & Medicaid Services Dialysis Facility Reports and Dialysis Facility Compare, which contain standardized information on all Medicare-certified dialysis facilities in the United States.

### Study Population

The study included all Medicare-certified dialysis facilities operating in the 50 US states during the study period. We excluded facilities lacking a valid Centers for Medicare & Medicaid Services certification number. Facilities were classified as providing PD services if they reported at least 1 PD patient treated as of December 31 of each survey year. For facilities that opened or closed during the study period, we included data only for the years they were operational, defined as having submitted complete annual facility reports to the Centers for Medicare & Medicaid Services.

### Primary Exposure

The primary exposure was calendar time, measured in years from 2010-2021. We analyzed both the absolute calendar year effect and year-over-year changes to capture both long-term trends and annual growth patterns.

### Outcome Measures

The primary outcome was mean PD census among facilities with active PD programs (defined as having ≥1 PD patient). Secondary outcomes included the following: (1) the proportion of facilities offering PD services and (2) annual growth rates in PD patient volume. PD census was defined as the number of PD patients treated in the facility on December 31 of each year.

### Covariates

We examined several facility-level characteristics as potential effect modifiers in our analyses. Geographic location was categorized according to US Census Bureau boundaries into the following 4 regions: (1) Northeast, (2) Midwest, (3) South, and (4) West. Urbanicity was classified using Rural-Urban Commuting Area codes (Table S1). We classified facilities based on organizational size into large dialysis organizations (LDOs), defined as in the kidney care choices model as organizations with >500 facilities per year, or small dialysis organizations (SDOs). Profit status was determined from Centers for Medicare & Medicaid Services facility surveys, which designate each center as either for-profit or nonprofit.

### Statistical Analysis

All analyses were conducted at the facility level. Descriptive statistics characterized trends in PD services, reporting the proportion of facilities with active PD programs and, among facilities with PD patients, the mean (standard deviation) and median (interquartile range) census over time. We used mixed-effects logistic regression with a random facility effect to assess the odds of a facility having at least 1 PD patient, and among facilities with active PD programs, we employed mixed-effects negative binomial regression with a random facility effect to analyze changes in PD census over time.

Both regression models were adjusted for facility characteristics including geographic region, organizational size (LDO vs SDO), and profit status (for-profit vs not-for-profit). To examine whether trends differed based on facility characteristics, we tested for interactions between time and each facility characteristic (organizational size, profit status, and region) in both models. Results are presented as odds ratios (OR) with 95% confidence intervals (CI) for the logistic models and incidence rate ratios (IRR) with 95% CI for the negative binomial models.

We conducted analyses comparing the final year (2021) with the baseline year (2010), with supplementary analyses examining year-to-year changes using time as a continuous variable. Statistical significance was set at *P* < 0.05 for main effects and interactions. Models accounted for the clustered nature of the data through random facility effects, allowing for correlation of observations within facilities over time while examining both cross-sectional and longitudinal trends in PD service provision.

All statistical analyses were performed using Stata 17 and 18 (StataCorp LLC). The study was deemed exempt from review by the Brown University Health Institutional Review Board because it analyzed publicly available administrative data reported at the facility level and did not involve any individual (identified or deidentified) patient information. Informed consent was not applicable as this study used publicly available, facility-level administrative data without any patient-level information.

## Results

**Figure 1:**
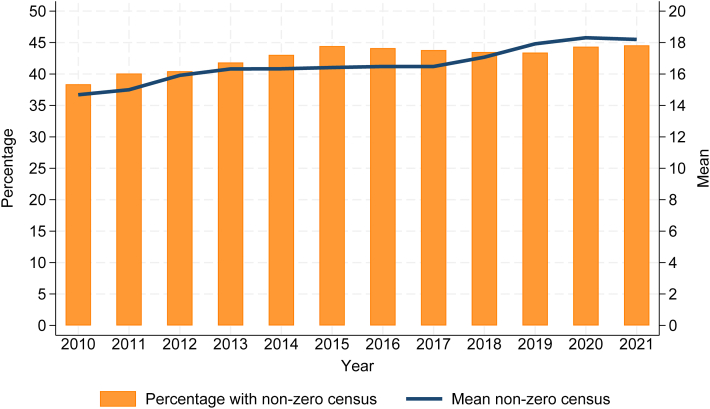
Peritoneal dialysis (PD) census over time. In adjusted analyses (Table 2) accounting for region, size, and profit status, facilities had 61% higher odds of offering PD services in 2021 compared with 2010 (OR, 1.61; 95% CI, 1.35-1.92). Among facilities with PD programs, the adjusted PD census increased by 50% over the study period (IRR, 1.50; 95% CI, 1.45-1.56). Abbreviations: CI, confidence interval; OR, odds ratio.

The study included 5,622 dialysis facilities in 2010, increasing to 7,904 facilities by 2021. In 2010, 38.4% of facilities had at least 1 PD patient, with a mean census of 14.7 (standard deviation 16.4) patients and a median of 10.0 (IQR, 4.0-19.0) patients among facilities offering PD. By 2021, the proportion of facilities with PD patients increased to 44.6%, with mean and median census rising to 18.2 (standard deviation 19.3) and 12.0 (IQR, 6.0-23.0) patients, respectively (Table 1, Table 2, Fig 1, and Table S2).

**Table 1:** Trends in PD Census

Year	No. of Centers	PD Patient Census		
		Percentage of Centers With At Least 1 PD Patient	Mean (SD)	Median (Q1-Q3)
2010	5,622	2,157 (38.4)	14.7 (16.4)	10.0 (4.0-19.0)
2011	5,761	2,309 (40.1)	15.0 (16.8)	10.0 (4.0-20.0)
2012	5,934	2,400 (40.4)	15.9 (18.0)	10.0 (5.0-20.0)
2013	6,141	2,569 (41.8)	16.3 (18.4)	11.0 (5.0-21.0)
2014	6,391	2,751 (43.0)	16.3 (18.5)	11.0 (5.0-21.0)
2015	6,562	2,916 (44.4)	16.4 (18.4)	11.0 (5.0-21.0)
2016	6,850	3,022 (44.1)	16.5 (17.8)	11.0 (5.0-21.0)
2017	7,128	3,122 (43.8)	16.5 (17.9)	11.0 (6.0-21.0)
2018	7,469	3,248 (43.5)	17.1 (18.7)	11.0 (6.0-21.5)
2019	7,702	3,343 (43.4)	17.9 (19.4)	12.0 (6.0-22.0)
2020	7,806	3,462 (44.4)	18.3 (19.7)	12.0 (7.0-23.0)
2021	7,904	3,522 (44.6)	18.2 (19.3)	12.0 (6.0-23.0)

Abbreviations: PD, peritoneal dialysis; SD, standard deviation.

Percentage calculations and descriptive statistics limited to facilities with at least 1 PD patient.

**Table 2:** Adjusted Models of Facility Growth

OR (95% CI) Per Y	IRR (95% CI) Per Y
Census > 0 2021 vs 2010	Mean Nonzero Census 2021 vs 2010
1.01 (1.002-1.02)	1.04 (1.03-1.04)
1.61 (1.35-1.92)	1.50 (1.45-1.56)

Abbreviations and definitions: CI, confidence interval; IRR, incidence rate ratio (negative binomial regression with random center effect); OR, odds ratio (logistic regression with random center effect).

Adjusted for region, size, for-profit status, and urbanicity.

The growth in PD services varied significantly based on facility characteristics (Table 3). Not-for-profit facilities showed the largest increase in PD program initiation (OR, 4.11; 95% CI, 2.53-6.68), whereas SDOs demonstrated stronger growth (OR, 3.01; 95% CI, 2.19-4.14) compared with LDOs (OR, 1.18; 95% CI, 0.96-1.45). Among facilities with active PD programs, for-profit facilities (IRR, 1.53; 95% CI, 1.47-1.58) and LDOs (IRR, 1.54; 95% CI, 1.47-1.61) showed greater census growth than their counterparts. Both organizational size and profit status showed significant interactions with time (*P* < 0.001 for both interactions) (Table S3).

**Table 3:** Facility-Level Factors Associated With PD Program Growth, 2010-2021

Size and Profit Status	OR (95% CI) for Census > 0 2021 vs 2010	OR (95% CI) Per Y	IRR (95% CI) for Mean Nonzero Census 2021 vs 2010	IRR (95% CI) Per Y
Organizational size				
LDO	1.18 (0.96-1.45)	0.98 (0.97-0.99)	1.54 (1.47-1.61)	1.039 (1.035-1.04)
SDO	3.01 (2.19-4.14)	1.11 (1.09-1.13)	1.40 (1.32-1.49)	1.03 (1.02-1.04)
*P* value for interaction	<0.001	<0.001	<0.001	0.007
Profit status				
For-profit	1.38 (1.15-1.65)	0.99 (0.98-1.01)	1.53 (1.47-1.58)	1.037 (1.03-1.04)
Not-for-profit	4.11 (2.53-6.68)	1.18 (1.14-1.22)	1.28 (1.17-1.41)	1.02 (1.01-1.03)
*P* value for interaction	<0.001	<0.001	<0.001	0.01
Region				
Northeast	1.47 (0.96-2.25)	1.02 (0.99-1.05)	1.50 (1.38-1.64)	1.04 (1.03-1.05)
Midwest	1.06 (0.73-1.53)	0.99 (0.97-1.01)	1.49 (1.38-1.60)	1.037 (1.03-1.04)
South	1.80 (1.40-2.32)	1.01 (0.99-1.02)	1.42 (1.34-1.49)	1.031 (1.03-1.04)
West	1.50 (1.004-2.25)	1.05 (1.02-1.07)	1.71 (1.58-1.85)	1.046 (1.04-1.05)
*P* value for interaction	0.13	0.008	0.09	0.008
Location				
Rural	11.5 (7.73-17.0)	1.21 (1.18-1.23)	1.60 (1.47-1.75)	1.04 (1.03-1.05)
Urban	0.96 (0.79-1.17)	0.97 (0.96-0.98)	1.49 (1.43-1.54)	1.035 (1.03-1.04)
*P* value for interaction	<0.001	<0.001	0.17	0.10

*Note*: Analysis limited to continental United States for geographic comparisons (unadjusted analyses).

Abbreviations and definitions: CI, confidence interval; IRR, incidence rate ratio (negative binomial regression with random center effect); LDO, large dialysis organization (defined as organization > 500 facilities per year); OR, odds ratio (logistic regression with random center effect); PD, peritoneal dialysis; SDO, small dialysis organization (all other dialysis organizations).

Regional variations were observed but were less pronounced (Table 3 and Table S4). The South showed the highest odds of new program initiation (OR, 1.80; 95% CI, 1.40-2.32), whereas the West demonstrated the largest increase in PD census among existing programs (IRR, 1.71; 95% CI, 1.58-1.85). The interaction between region and time was not statistically significant for either program initiation (*P* = 0.13) or census growth (*P* = 0.09).

Notable differences emerged between rural and urban facilities. Rural facilities demonstrated dramatically higher odds of initiating new PD programs (OR, 11.5; 95% CI, 7.73-17.0) compared with urban facilities (OR, 0.96; 95% CI, 0.79-1.17), with a significant interaction with time (*P* < 0.001). However, among facilities with existing PD programs, census growth was similar between rural and urban settings (IRR, 1.60 vs 1.49, respectively), with no significant interaction with time (*P* = 0.17) (Table S5).

## Discussion

Our study provides novel insights into these facility-level dynamics underlying the recent expansion of PD in the United States from 2010-2021. Although prior research has established that PD use has increased nationally, our analysis addresses the critical question of whether this growth has occurred primarily through the establishment of new, smaller PD programs or through expansion of existing programs.^2^ Our findings reveal a nuanced picture, which was as follows: both mechanisms have contributed substantially to overall PD growth, with the proportion of facilities offering PD services increasing from 38.4%-44.6% and the mean census among active PD programs rising from 14.7-18.2 patients. These results indicate meaningful progress toward expanding PD access at a national level, consistent with policy efforts aimed at promoting home dialysis modalities.

We found that these growth patterns varied significantly based on organizational characteristics. SDOs exhibited marked growth in initiating PD services, whereas LDOs showed greater expansion in PD census among facilities already offering PD. Similarly, nonprofit facilities were more likely to start new PD programs, whereas for-profit facilities demonstrated greater growth in PD patient volume. It should be noted that these categories have significant overlap. These differences suggest distinct strategic approaches to PD expansion. Nonprofit and smaller organizations may have prioritized initial PD program establishment, potentially driven by mission-oriented goals or local patient demand. In contrast, for-profit and larger organizations may have emphasized expanding existing programs, leveraging economies of scale and established infrastructure to efficiently increase patient census.

The decline in the number of SDO and nonprofit facilities over the study period likely reflects consolidation within the dialysis sector rather than contraction specific to PD programs. This consolidation may partly explain the apparent differences between LDO and SDO growth patterns, as the denominator of SDO and nonprofit facilities decreased over time. It is also possible that for-profit entities preferentially acquired hemodialysis-only facilities rather than facilities with PD capability, which could further contribute to the observed patterns of PD program initiation across organizational types.

Regional variations were evident but less pronounced, with the South showing the highest odds of new PD program initiation and the West experiencing the most substantial growth in PD patient census. These findings are consistent with prior work showing growth in PD supply in the South, Midwest, and West.^16^^,^^17^ These findings highlight the potential role of geographic factors such as organizational strategy, competition, regional policy environments, local health care infrastructure, demographic shifts, and physician practice patterns in influencing PD growth trajectories.

These findings add to several patient-level factors that are associated with the selection and increasing use of PD. Younger age has historically linked to PD choice, although recent growth (2009-2019) has been proportionally greater among older patients (75+ years), reducing age-related disparities.^6^^,^^8^ Clinically, having fewer comorbid conditions and lower serum albumin has been associated with PD selection.^8^ Racial and ethnic disparities are significant; Black and Hispanic patients are consistently less likely than White patients to initiate PD, with these disparities being most pronounced among younger adults.^18^, ^19^, ^20^ Although recent trends show gap narrowing for Hispanic individuals compared with White individuals, no similar reduction was observed for Black patients.^6^ Socioeconomic factors play a role, partially explaining lower PD use among Black and Hispanic patients; factors such as employment, marriage, higher education, greater patient autonomy, and living in socioeconomically advantaged areas are associated with higher PD uptake.^6^^,^^8^^,^^19^ In addition, earlier and more frequent prekidney failure nephrology care is associated with increased PD use, and the early coronavirus disease-2019 pandemic saw a temporary increase in PD initiation odds.^8^

Our findings have important implications for policy and practice. The observed growth patterns reveal differential expansion strategies across facility types, with nonprofit and smaller organizations prioritizing new program establishment, whereas for-profit and larger organizations focused on expanding existing programs. These stark differences suggest that organizational characteristics significantly influence approaches to PD growth. However, the stark differences in growth patterns across facility types indicate that these policies may have differential effects depending on organizational characteristics. In addition, the slower growth in program initiation compared with census expansion among existing programs suggests that barriers to starting new PD programs may be more significant than barriers to growing existing ones. Future policy efforts might benefit from targeted approaches that recognize and address the distinct challenges and opportunities faced by different types of dialysis providers.

Several limitations should be considered when interpreting our results. Our analysis relied on facility-level aggregated data, which precluded examination of patient-level factors influencing program size and growth. Future analyses could incorporate facility-level patient case-mix indicators available in dialysis facility reports (eg, percent of patients with diabetes or hypertension) as well as linked area-level sociodemographic characteristics. Such extensions would help clarify whether PD program growth is occurring in communities with greater clinical or social vulnerability. Second, the cross-sectional nature of our analysis at yearly intervals may not capture more nuanced temporal trends or seasonal variations in PD use. Furthermore, our study period ended in 2021, limiting our ability to evaluate the full impact of the end-stage renal disease treatment choices Model implemented in 2021 and of the coronavirus disease-2019 pandemic, both of which have likely led to further changes in PD use.

In conclusion, our study demonstrates that the growth in PD use from 2010-2021 has occurred through both the establishment of new programs and the expansion of existing ones, with important variations based on organizational type and geographic region.
