# Supplementary material for: The Changing Landscape of Peritoneal Dialysis in America: A Facility-Level Analysis of Growth
Source: Kidney Med. 2026 May 8;8(7):101391. doi: 10.1016/j.xkme.2026.101391 (PMC13312188; doi:10.1016/j.xkme.2026.101391)
Supplement: Supplementary File (PDF) — Tables S1-S5 [file mmc1.pdf]

Table S1. RUCA Code Classifications

Category	Values
Urban	1.0, 1.1, 2.0, 2.1, 4.1, 5.1, 7.1, 8.1, 10.1
Rural	3.0, 4.0, 4.2, 5.0, 5.2, 6.0, 6.1, 7.0, 7.2, 7.3, 7.4, 8.0, 8.2, 8.3, 8.4, 9.0, 9.1, 9.2, 10.0, 10.2, 10.3, 10.4, 10.5, 10.6

Classification system developed by the US Department of Agriculture Economic Research Service

Table S2. Facility Characteristics by Year

	Available data for at least 1 years		
	**Total**	**2010**	**2021**
N	8,982	6,350	8,185
Facility profit status			
For profit	7,491 (83.4%)	5,325 (83.9%)	7,265 (88.8%)
Non-profit	1,435 (16.0%)	1,022 (16.1%)	919 (11.2%)
Unavailable	56 (0.6%)	3 (0.0%)	1 (0.0%)
Large Dialysis Organization			
No	3,623 (40.3%)	2,578 (40.6%)	2,156 (26.3%)
Yes	5,359 (59.7%)	3,772 (59.4%)	6,029 (73.7%)
Census regions			
Northeast	1,343 (15.0%)	910 (14.3%)	1,137 (13.9%)
Midwest	2,079 (23.1%)	1,514 (23.8%)	1,822 (22.3%)
South	3,934 (43.8%)	2,745 (43.2%)	3,718 (45.4%)
West	1,549 (17.2%)	1,126 (17.7%)	1,444 (17.6%)
PR & Territories	77 (0.9%)	55 (0.9%)	64 (0.8%)
Provider network			
1)CT,ME,MA,NH,RI,VT	250 (2.8%)	189 (3.0%)	211 (2.6%)
2)NY	446 (5.0%)	276 (4.3%)	365 (4.5%)
3)NJ,PR,VI	310 (3.5%)	200 (3.1%)	267 (3.3%)
4)DE,PA	438 (4.9%)	315 (5.0%)	382 (4.7%)
5)VA,WV,MD,DC	518 (5.8%)	346 (5.4%)	476 (5.8%)
6)GA,NC,SC	882 (9.8%)	636 (10.0%)	834 (10.2%)
7)FL	580 (6.5%)	384 (6.0%)	549 (6.7%)
8)AL,MS,TN	521 (5.8%)	403 (6.3%)	504 (6.2%)
9)IN,KY,OH	748 (8.3%)	558 (8.8%)	678 (8.3%)
10)IL	398 (4.4%)	272 (4.3%)	365 (4.5%)
11)MN,MI,ND,SD,WI	653 (7.3%)	478 (7.5%)	552 (6.7%)
12)IA,KS,MO, NE	422 (4.7%)	318 (5.0%)	360 (4.4%)
13)AR,LA,OK	399 (4.4%)	304 (4.8%)	368 (4.5%)
14)TX	855 (9.5%)	537 (8.5%)	820 (10.0%)

	Available data for at least 1 years		
	Total	2010	2021
N	8,982	6,350	8,185
15)AZ,CO,NV,NM,UT,WY	431 (4.8%)	327 (5.1%)	396 (4.8%)
16)AK,ID,MT,OR,WA	280 (3.1%)	189 (3.0%)	250 (3.1%)
17)AS,GU,MP,HI,N.CA	365 (4.1%)	267 (4.2%)	340 (4.2%)
18)S.CA	486 (5.4%)	351 (5.5%)	468 (5.7%)

Data includes all facilities with available data for at least one year during study period
Percentages calculated among facilities with available data for each characteristic

Table S3. Trends stratified by region

	Northeast			Midwest			South			West		
Year	N centers	> 0 PD patients	Mean (SD) if > 0	N centers	> 0 PD patients	Mean (SD) if > 0	N centers	> 0 PD patients	Mean (SD) if > 0	N centers	> 0 PD patients	Mean (SD) if > 0
2010	792	356 (44.9%)	10.9 (12.0)	1,324	500 (37.8%)	12.4 (12.5)	2,457	867 (35.3%)	15.4 (16.8)	999	408 (40.8%)	19.1 (21.4)
2011	812	379 (46.7%)	11.0 (12.0)	1,352	520 (38.5%)	12.8 (12.4)	2,521	962 (38.2%)	15.4 (16.9)	1,025	424 (41.4%)	20.2 (22.8)
2012	832	383 (46.0%)	11.7 (12.4)	1,395	554 (39.7%)	13.6 (13.0)	2,597	998 (38.4%)	16.3 (17.4)	1,059	442 (41.7%)	21.5 (25.7)
2013	856	415 (48.5%)	11.9 (12.9)	1,439	582 (40.4%)	14.2 (13.4)	2,690	1,082 (40.2%)	16.5 (17.5)	1,102	467 (42.4%)	22.6 (26.7)
2014	872	434 (49.8%)	11.8 (12.9)	1,503	626 (41.7%)	14.0 (13.3)	2,811	1,177 (41.9%)	16.3 (17.4)	1,150	491 (42.7%)	23.2 (27.4)
2015	899	454 (50.5%)	11.7 (12.8)	1,527	653 (42.8%)	14.5 (13.6)	2,909	1,264 (43.5%)	16.1 (17.5)	1,169	520 (44.5%)	23.6 (26.3)
2016	935	468 (50.1%)	11.9 (12.2)	1,593	681 (42.7%)	14.6 (13.6)	3,057	1,306 (42.7%)	16.2 (17.1)	1,208	542 (44.9%)	23.4 (24.8)
2017	977	477 (48.8%)	11.7 (11.7)	1,633	700 (42.9%)	14.5 (13.3)	3,206	1,354 (42.2%)	16.2 (16.8)	1,252	567 (45.3%)	23.6 (25.9)
2018	1,014	497 (49.0%)	12.3 (12.1)	1,713	724 (42.3%)	15.2 (13.9)	3,381	1,414 (41.8%)	16.6 (17.1)	1,299	586 (45.1%)	24.6 (28.1)
2019	1,058	508 (48.0%)	12.9 (12.2)	1,739	736 (42.3%)	16.0 (14.2)	3,490	1,457 (41.7%)	17.3 (17.6)	1,354	615 (45.4%)	25.8 (29.4)
2020	1,084	530 (48.9%)	13.3 (13.1)	1,719	741 (43.1%)	16.6 (14.3)	3,544	1,524 (43.0%)	17.5 (17.7)	1,397	640 (45.8%)	26.4 (29.7)
2021	1,094	533 (48.7%)	13.5 (12.7)	1,742	754 (43.3%)	16.1 (14.1)	3,594	1,557 (43.3%)	17.4 (17.3)	1,412	649 (46.0%)	26.6 (29.1)

Table S4. Trends stratified by LDO or for-profit status

	LDO			SDO			For profit			Non-profit		
Year	N centers	> 0 PD patients	Mean (SD) if > 0	N centers	> 0 PD patients	Mean (SD) if > 0	N centers	> 0 PD patients	Mean (SD) if > 0	N centers	> 0 PD patients	Mean (SD) if > 0
2010	3,321	1,266 (38.1%)	14.0 (14.6)	2,301	891 (38.7%)	15.6 (18.6)	4,658	1,790 (38.4%)	14.4 (15.8)	961	365 (38.0%)	15.9 (18.9)
2011	3,485	1,412 (40.5%)	14.2 (15.0)	2,276	897 (39.4%)	16.2 (19.3)	4,816	1,952 (40.5%)	14.7 (16.4)	943	357 (37.9%)	16.5 (18.9)
2012	3,692	1,503 (40.7%)	15.3 (15.9)	2,242	897 (40.0%)	17.0 (20.9)	5,033	2,061 (40.9%)	15.6 (17.5)	900	339 (37.7%)	17.7 (20.2)
2013	3,973	1,671 (42.1%)	15.8 (17.4)	2,168	898 (41.4%)	17.3 (20.2)	5,242	2,232 (42.6%)	16.0 (17.9)	899	337 (37.5%)	18.5 (21.6)
2014	4,552	1,960 (43.1%)	15.9 (17.7)	1,839	791 (43.0%)	17.4 (20.5)	5,504	2,410 (43.8%)	15.9 (17.6)	887	341 (38.4%)	19.1 (23.8)
2015	4,694	2,068 (44.1%)	15.9 (17.3)	1,868	848 (45.4%)	17.6 (20.7)	5,685	2,552 (44.9%)	16.0 (17.4)	877	364 (41.5%)	19.5 (23.9)
2016	4,907	2,119 (43.2%)	16.0 (16.7)	1,943	903 (46.5%)	17.5 (20.1)	5,964	2,654 (44.5%)	16.0 (16.7)	886	368 (41.5%)	20.0 (23.8)
2017	5,203	2,232 (42.9%)	16.2 (16.7)	1,925	890 (46.2%)	17.2 (20.5)	6,258	2,747 (43.9%)	15.9 (16.6)	869	374 (43.0%)	20.6 (25.1)
2018	5,580	2,375 (42.6%)	17.1 (17.8)	1,889	873 (46.2%)	17.1 (21.0)	6,591	2,869 (43.5%)	16.5 (17.4)	877	378 (43.1%)	21.1 (26.4)
2019	5,763	2,439 (42.3%)	18.1 (18.7)	1,939	904 (46.6%)	17.5 (21.2)	6,830	2,964 (43.4%)	17.4 (18.2)	871	379 (43.5%)	21.6 (26.8)
2020	5,829	2,513 (43.1%)	18.5 (19.1)	1,977	949 (48.0%)	17.7 (21.2)	6,936	3,069 (44.2%)	17.9 (18.5)	870	393 (45.2%)	21.5 (27.1)
2021	5,867	2,535 (43.2%)	18.6 (18.9)	2,037	987 (48.5%)	17.3 (20.3)	7,047	3,126 (44.4%)	17.9 (18.2)	857	396 (46.2%)	20.5 (26.1)

Table S5. Trends stratified by urbanicity

	Rural			Urban		
Year	N centers	> 0 PD patients	Mean (SD) if > 0	N centers	> 0 PD patients	Mean (SD) if > 0
2010	1,119	322 (28.8)	8.0 (8.7)	4,104	1,725 (42.0)	16.0 (17.3)
2011	1,158	364 (31.4)	8.2 (9.0)	4,287	1,859 (43.4)	16.4 (17.8)
2012	1,218	404 (33.2)	9.0 (9.2)	4,504	1,946 (43.2)	17.5 (19.1)
2013	1,248	448 (35.9)	9.0 (8.7)	4,700	2,074 (44.1)	18.0 (19.6)
2014	1,289	497 (38.6)	8.8 (8.6)	4,962	2,226 (44.9)	18.1 (19.8)
2015	1,332	541 (40.6)	8.7 (8.5)	5,155	2,360 (45.8)	18.2 (19.6)
2016	1,369	567 (41.4)	8.9 (8.4)	5,441	2,448 (45.0)	18.2 (18.9)
2017	1,402	574 (40.9)	9.2 (8.7)	5,675	2,534 (44.7)	18.1 (19.0)
2018	1,428	590 (41.3)	9.8 (9.2)	6,018	2,655 (44.1)	18.7 (19.9)
2019	1,441	602 (41.8)	10.3 (9.6)	6,247	2,739 (43.8)	19.6 (20.6)
2020	1,423	621 (43.6)	10.8 (9.4)	6,368	2,838 (44.6)	19.9 (21.0)
2021	1,434	634 (44.2)	10.6 (9.7)	6,451	2,886 (44.7)	19.9 (20.4)
